# Reconstruction of a Complex Metacarpal Shaft Fracture With Segmental Bone Loss Using Autologous Iliac Crest Bone Graft

**Published:** 2015-08-20

**Authors:** Aditya Sood, Stella Chung, Paul J. Therattil, Edward S. Lee

**Affiliations:** Division of Plastic and Reconstructive Surgery, Department of Surgery, Rutgers University–New Jersey Medical School, Newark

**Keywords:** metacarpal shaft fracture, segmental bone loss, iliac crest bone graft, bone grafts, comminuted hand fractures

**Figure:**
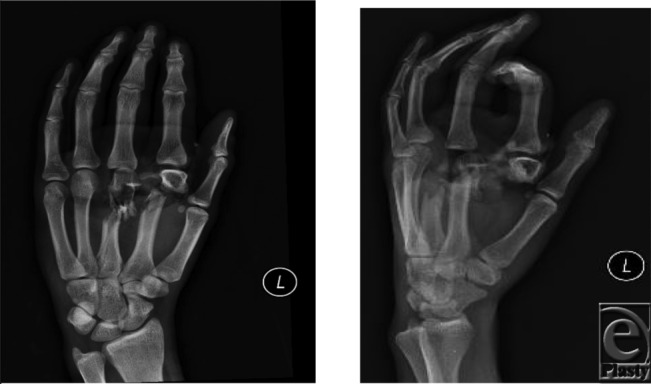


## DESCRIPTION

A 22-year-old man, right-hand dominant, presented with pain in his left hand after a severe crush injury at work using a forklift. Deformity, open fractures, swelling over the dorsum of his hand, and paresthesias to the index and middle digits were evident during initial examination. He was vascularly intact. Radiographs showed markedly comminuted fractures of the index metacarpal neck and middle metacarpal shaft with radial displacement and segmental bone loss.

## QUESTIONS

1. **What are the indications and treatment options for complex comminuted metacarpal fractures with significant bone loss?**
2. **When is the best time for bone grafting?**
3. **What are the benefits of using iliac crest bone grafts as a donor site?**
4. **What are the complications of iliac crest bone grafting for metacarpal defects?**

## DISCUSSION

About 5% to 10% of hand fractures may a have bone defect that requires filling with autologous bone graft or bone substitute.^1^ Bone grafts have osteogenic, osteoinductive, and osteoconductive properties that induce healing. In segmental loss of the metacarpals, early grafting and internal fixation are crucial for recovery of motion. Augmentation with autologous bone graft should be considered for defects greater than one half the diameter of the diaphysis or one half the diameter of a major unsupported articular fragment.^2^ Common donor sites are iliac crest, rib, distal radius, and fibula. Olecranon is an alternative source of corticocancellous graft.^2^ Among these, iliac crest has the most cortical component. For large skin defects, one can consider free fascial and regional pedicled flaps.

**Figure 1:**
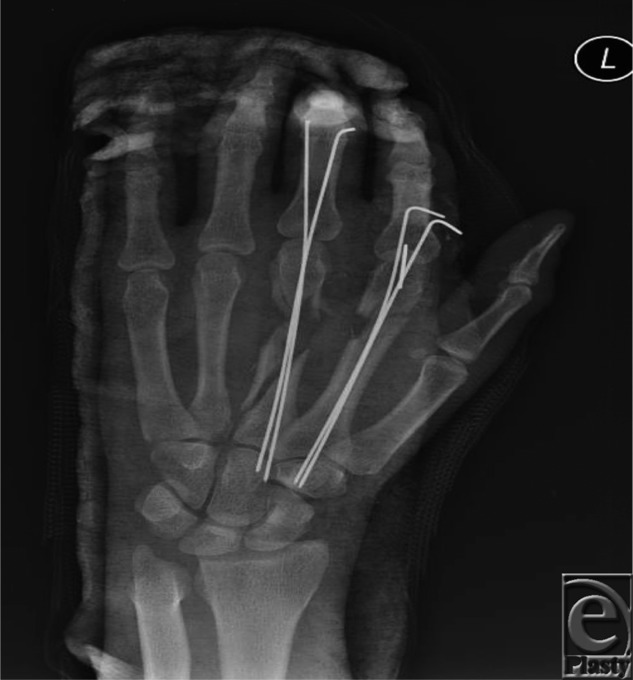
Because of concern for infection secondary to debris and soft-tissue viability, a staged reconstruction was undertaken. Initial treatment included a thorough washout and debridement, followed by K-wire fixation of the metacarpal bones to preserve length.

Segmental loss of the metacarpal shaft is frequently associated with open injury and soft-tissue defects.^3^ Radical and thorough debridement of the wound is the first critical step of treatment. Length must be maintained for osseous stability with bone graft and internal fixation within 10 days (Fig 1).^2^ In a recent review, immediate reconstruction with corticocancellous bone graft has been shown expedited active range of motion, aggressive therapy, decreased morbidity, and less hospital cost.^4^ However, if concern for infection is high, staged reconstruction must be performed after debridement and primary soft-tissue coverage.

**Figure 2:**
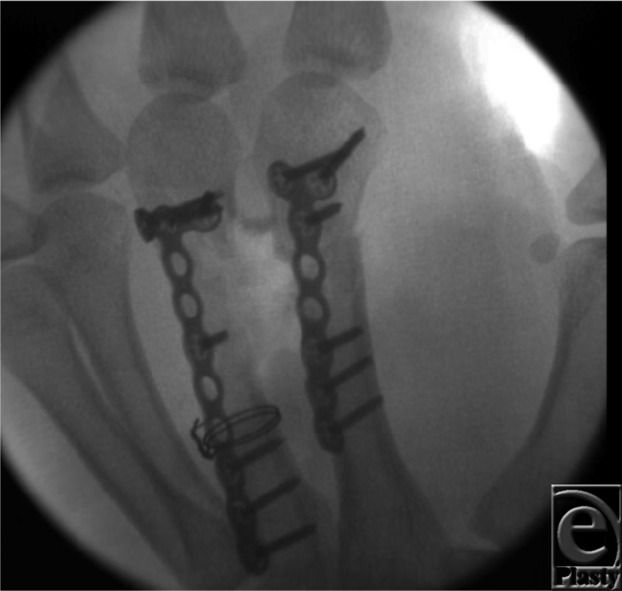
One week after initial stabilization and determining viability of the soft tissues and clearance of infection, iliac crest corticocancellous bone graft was harvested and fixated to the index and middle metacarpals.

The iliac crest is a preferred donor site for corticocancellous or cancellous graft to stabilize bone fragments in the hand (Fig 2). Iliac crest is advantageous for extensive defects because a large volume of cancellous bone can be obtained. Iliac crest bone graft may also be used for metacarpal tumors and may be used as a vascularized bone graft for carpal defects.^5^^,^^6^ A significant amount of soft tissue can be raised with bone and may be used as a combined osteomusculocutaneous flap, receiving vascular input from superficial and deep circumflex iliac vessels that supply blood to the bone graft reconstruction.

Severity of the injury, rather than implant selection, is correlated with complications.^7^ The most common complication of comminuted open fractures is stiffness secondary to tendon and capsular adhesions.^7^ Other complications include nonunion/malunion, bone loss, and wound-healing problems. Donor site morbidities are hematoma, pain, lateral femoral cutaneous nerve injury, avulsion fractures of the anterior superior iliac spine, and hernia. Temporary reflex sympathetic dystrophy may be seen. Early detection, stellate ganglion blocks, neurosuppressive medications, and an intense therapy program are most beneficial to prevent complications.^8^

Preoperative planning includes characterizing the bony defect, soft-tissue loss, clearing the debris/necrotic tissues and removing potential sites of infection, and determining patency of recipient vessels if indicated. Radical debridement of affected bone and soft tissue is essential before grafting. Stabilization of fracture-bone graft complex leads to unimpeded revascularization of the bone graft and subsequent healing. Physical therapy in the postoperative period helps minimize the effects of posttraumatic arthritis.
